# Bacterial Surface-Displayed GII.4 Human Norovirus Capsid Proteins Bound to HBGA-Like Molecules in Romaine Lettuce

**DOI:** 10.3389/fmicb.2017.00251

**Published:** 2017-02-20

**Authors:** Ming Wang, Shaofeng Rong, Peng Tian, Yue Zhou, Shimin Guan, Qianqian Li, Dapeng Wang

**Affiliations:** ^1^Department of Bioengineering, Shanghai Institute of TechnologyShanghai, China; ^2^Produce Safety and Microbiology Research Unit, Western Regional Research Center, Agricultural Research Service – United States Department of Agriculture, AlbanyCA, USA; ^3^MOST-USDA Joint Research Center for Food Safety, School of Agriculture and Biology, Shanghai Jiao Tong UniversityShanghai, China

**Keywords:** norovirus, cell surface display, capsid protein, protruding domain, HBGAs, romaine lettuce, GII.4

## Abstract

Human Noroviruses (HuNoVs) are the main cause of non-bacterial gastroenteritis. Contaminated produce is a main vehicle for dissemination of HuNoVs. In this study, we used an ice nucleation protein mediated surface display system to present the protruding domain of GII.4 HuNoV capsid protein on bacterial surface and used it as a new strategy to explore interaction between HuNoV protein and receptor candidates from romaine lettuce. The surface-displayed HuNoV proteins were confirmed on the surface of the transformed bacteria by an immunofluorescence assay. The distribution patterns of the surface-displayed HuNoV proteins in romaine lettuce were identified through a confocal immunofluorescence assay. The surface-displayed HuNoV proteins could be found in the stomata, and the surfaces of vein and leaf of romaine lettuce. The surface-displayed HuNoV proteins could be captured by an ELISA assay utilizing extract from leaf (LE) or vein (VE). The binding of the surface-displayed HuNoV proteins to LE or VE could be competitively blocked by histo-blood group antigens from human saliva. In addition, the binding of the surface-displayed HuNoV proteins to LE or VE could also be attenuated by heat denaturation of lettuce proteins, and abolished by oxidation of lettuce carbohydrates. The results indicated that histo-blood group antigen-like molecules in LE or VE were involved in the binding of the surface-displayed HuNoV proteins to romaine lettuce. All data demonstrated that the surface-displayed HuNoV proteins could be utilized in a new and simple system for investigation of the interaction between the HuNoVs and their candidate ligands.

## Introduction

Norovirus (NoV) is a single-stranded RNA virus of the family *Caliciviridae*. Based on the sequence of the major capsid protein (VP1), noroviruses have been categorized into seven genogroups (GI though GVII) (Vinjé, 2015). The GI, GII, and GIV genogroups are capable of infecting humans, and comprise the human noroviruses (HuNoVs). GII strains are more common, and are the main cause of human non-bacterial gastroenteritis worldwide (Hoa Tran et al., 2013).

Infection by HuNoVs could be associated with the consumption of fresh produce, such as romaine lettuce (Ethelberg et al., 2010), onion (Dicaprio et al., 2015), strawberries (Made et al., 2013), and raspberries (Sarvikivi et al., 2012). Produce could be contaminated before harvest by irrigation water (Mok et al., 2014), manure or bio-solids (Wei et al., 2010). Harvest and post-harvest cross-contamination of produce could result from human contact, including handling, chopping/slicing and mixing (Grove et al., 2015). In addition, HuNoVs are stable in the environment and remain infectious in fresh produce surface for a long time (Verhaelen et al., 2012). Therefore, contaminated produce becomes one of the important vehicles for the transmitting of HuNoVs.

Until recent reports of successful replication of HuNoVs in human B cells (Jones et al., 2015) and stem cell-derived human enteroids (Ettayebi et al., 2016), the lack of an *in vitro* cultivation system had been a major barrier to the study of HuNoVs. In lieu of HuNoVs, viral-like particles (VLPs) and surrogate viruses including feline Calicivirus (FCV), murine norovirus (MNV), Tulane virus (TV) have been used (Jiang et al., 1992; Bozkurt et al., 2013; Xu et al., 2015). Limited literatures were available on interactions between HuNoVs and ligands from produce, and results were not consistent. GII.4 HuNoV, TV, and MNV were found to internalize into lettuce *via* roots, and disseminate into shoots and leaves (Dicaprio et al., 2012). Wei et al reported that MNV were found on leaf surfaces, in stomata, and at cut edges of romaine lettuce (Wei et al., 2010). Wang et al reported that porcine sapovirus could attach to romaine lettuce and remain infectious for a week after storage at refrigeration temperatures (Wang et al., 2012). Gandhi et al reported that GI.1 VLPs could be found in clusters along romaine lettuce veins. The binding of GI VLPs to romaine extract (RE) could not be competitively inhibited by porcine gastric mucin (PGM), suggesting that molecules involved in the binding of GI NoV to romaine lettuce might not be related to PGM-like carbohydrates (Gandhi et al., 2010). Esseili et al. (2012) reported that GII.4 VLPs bound to carbohydrates of romaine lettuce leaves. They demonstrated that attachment of GII.4 VLPs to young leaves was primarily associated with proteins whereas for older leaves it was primarily through carbohydrates. However, the binding of VLPs to romaine lettuce leaf cell wall materials could only be partially inhibited by PGM, carbohydrate-binding lectins, and oxidation of carbohydrate. Gao et al. (2016) reported that binding of GII.4 VLPs to romaine lettuce was enhanced after digesting lettuce leaves with cell-wall-degrading enzymes. They further demonstrated that HBGA-like molecules exist within lettuce tissue, and GII.4 VLPs could bind the exposed fucose moiety of HBGAs.

Most studies of the interaction between HuNoVs and receptor candidates on/in romaine lettuce utilized recombinant HuNoV VLPs expressed from eukaryotic expression systems, or P-particles expressed from prokaryotic systems (Tan and Jiang, 2005). It has been well documented that there was no difference between eukaryote-expressed recombinant proteins (VLP) and prokaryote-expressed proteins (P particles) in its biological functions such as HBGA binding and immunogenicity (Tan et al., 2008, 2011; Tamminen et al., 2012). However, there are some technical issues on making them. Making recombinant eukaryotic system was complicated and time-consuming. Expression in *Escherichia coli* had downstream processing issues due to the presence of inclusion bodies which need to be disrupted to form functional virions by complicated purification steps. The purpose of the study was to develop a new strategy to present noroviral proteins on the surface of the transformed *E. coli* to avoid complicated purification steps and use this new system to identify candidate receptors for HuNoV binding in lettuce.

## Materials and Methods

### Plasmid DNA and Bacteria Transformation

**FIGURE 1:**
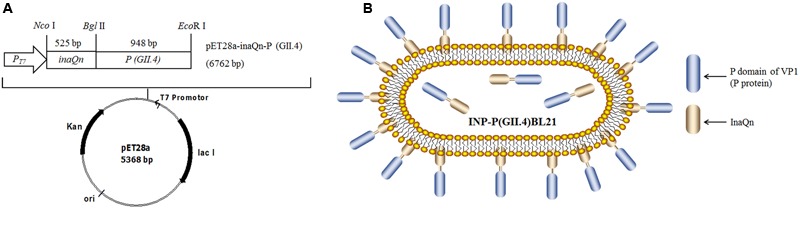
**(A)** Plasmid DNA map; **(B)** Schematic figure for SD-GII.4P displayed on the surface of pET28a-inaQn-P (GII.4) transformed bacteria [INP-P (GII.4) BL21]. *inaQn*: N-terminal of ice nucleation protein encoding gene; *P(GII.4)*: P domain of GII.4 HuNoVs capsid protein encoding gene; *P_T7_*: T7 promoter; *Nco* I, *Bgl* II and *Eco*R I: restriction enzymes; Kan: kanamycin - resistant gene; ori: pBR322 origin; lac I: overexpressed lac repressor.

*Escherichia coli* BL21 (Thermo Fisher, Shanghai, China) was used as competent cell for recombinant plasmid transformation and expression of the target protein. pET28a-inaQn-P (GII.4) and pET28a-P (GII.4) were constructed as previously described (Niu et al., 2015), pET28a-inaQn was constructed by inserting *inaQn* into *Nco* I/*Bam*H I digested pET28a and used as a control. All the recombinant plasmids were used to transform bacteria *E. coli* BL21. **Figures 1A,B** presented for the plasmid DNA map and a schematic figure for displayed P protein on the surface of pET28a-inaQn-P(GII.4) transformed bacteria [INP-P (GII.4) BL21].

### Culture and Expression of SD-GII.4P Fusion Protein in *E. coli* BL21

Recombinant *E. coli* BL21 harboring *inaQn* and *P protein* fusion gene [named INP-P (GII.4) BL21], *P protein* gene [named P (GII.4) BL21] and *inaQn* gene (named INP BL21) were cultured as previously reported (Niu et al., 2015). Cells were cultured in 5.0 ml of LB medium containing 100 μg/ml kanamycin with shaking (220 rpm) at 37°C, overnight. The cells (50.0 μl) were subcultured in 5.0 ml fresh LB medium with 60 μg/ml kanamycin. When the OD_600_ reached 0.5, isopropyl-β-D-thiogalactopyrano-side (IPTG) was added to reach a final concentration of 0.4 mmol/l. The cells were incubated at 25°C for 12 h, then washed and diluted to OD_600_ = 1.0 with sterile PBS, and then kept at 4°C for further use.

### Identification of Surface Displayed P Proteins by Immunofluorescence Assay

The localization of surface-displayed P protein (SD-GII.4P) was examined using immunofluorescence microscopy (Olympus, Japan) as previously described (Li et al., 2009). Anti-VP1 (GII.4) polyclonal antisera from immunized Balb/C mice (1:5000 in PBS) were used as the primary antibody (Niu et al., 2015), and 1:100-diluted FITC-conjugated goat anti-mouse IgG (Thermo Fisher, USA) was used as the secondary antibody. The anti-VP1 (GII.4) polyclonal antisera were pre-absorbed with *E. coli* BL21 extract to reduce potential non-specific. Three sets of negative controls were used. P (GII.4) BL21, INP BL21, and *E. coli* BL21 were used to test non-specific binding of polyclonal antisera. INP-P (GII.4) BL21 without primary antibody (with PBS only) was used as additional negative control for non-specific binding of FITC-conjugated secondary antibody.

### Preparation of Romaine Lettuce

Romaine lettuce was collected randomly from a local grocery store and stored at 4°C. The leaves were washed three times with sterile distilled water. For microscopy, approximately 2 cm^2^ of the strips were cut from the ends of leaves using a sterile blade. For ELISA, the romaine lettuce was made into leaf extract (LE) and vein extract (VE), collectively referred to as RE. LE was prepared from the top half of a romaine leaf (15 g), while VE was prepared from the excised main veins. The same volume of PBS (wt/vol) was added to each vegetable matter sample, and blended for 5 min at 4°C. The liquid phase was transferred to a sterile microcentrifuge tube and centrifuged at 10,000 × *g* for 10 min at 4°C. The supernatant was transferred to a fresh sterile tube and kept at 4°C for further use.

### Preparation of Boiled Saliva

Human saliva was collected from A, B, O blood type volunteers and treated according to the previous report, with minor modification (Wang et al., 2014). The secretor status of individual saliva was indirectly determined by an ELISA assay for its ability to bind purified bacteria-expressed GI and GII VP1 and P domains (Niu et al., 2015) and directly determined with corresponding monoclonal antibodies. Although HuNoV binding ability was confirmed in all saliva tested, three saliva samples from the same blood type were mixed and used for competitively assays. The study was approved by the Institutional Bio-safety Committees (IBC) of College of Agriculture and Biology, Shanghai Jiao Tong University, and written informed consent was obtained from the volunteers. Briefly, each type of saliva was collected from at least three volunteers and mixed. Then, each saliva sample was boiled for 5 min, and then followed with centrifugation at 10,000 × *g* for 5 min at 4°C. The individual supernatant of saliva from each blood group was mixed, aliquoted and stored at –20°C for further use. Saliva from non-secretors was used as negative control.

### Identification of SD-GII.4P Binding to Romaine Lettuce by Confocal Immunofluorescence Assay

Strips of romaine lettuce on glass slides were blocked with 1% bovine serum albumin (BSA, Yeasen, Shanghai, China) in PBS at 4°C, overnight, and then soaked in sterile PBS for 5 min. The strips were moved into a sterile plate containing INP-P (GII.4) BL21 or *E. coli* BL21 (negative control), and incubated at room temperature for 1 h. The strips were then washed with PBS solution and put on a slide. Then, 70 μl of primary antibody (Niu et al., 2015) at a dilution of 1:2,000 in PBS was added onto the surface of strips and incubated at room temperature for 1 h. After incubation, strips were washed three times with PBS. A volume of 50 μl FITC-conjugated goat anti-mouse IgG (H+L) (Yeasen, Shanghai, China) at a dilution of 1:100 in PBS was added onto the strips, and incubated at room temperature for 1 h. After washing 3 times with PBS, 30 μl PBS was applied to the surface of the romaine, covered with a coverslip, and observed under a confocal laser scanning microscope (Leica Microsystems, Wetzlar, Germany). The images were magnified at 700×.

### Romaine Lettuce Extract-Binding or PGM-Binding Based ELISA

The RE-binding enzyme-linked immunosorbent assay (ELISA) was performed as previously reported (Gandhi et al., 2010), with minor modification. Briefly, LE or VE was diluted in PBS (1:5, pH = 7.4) (Gandhi et al., 2010), and PGM (Sigma, MI, USA) was dissolved in carbonate buffer solution (pH = 9.6) to 1.0 mg/ml (Wang et al., 2014). Then, LE, VE, or PGM was added into and allowed to coat 96-well microtiter plates (100 μl per well) at 4°C overnight. Plates were washed three times with PBS, and blocked with 1% BSA at 37°C for 2 h. Plates were washed three times with PBS containing 0.1% Tween-20 (PBS-T). The INP-P (GII.4) BL21, *E. coli* BL21, P (GII.4) BL21, and INP BL21 samples (100 μl) were added to each well, respectively, and incubated at 37°C for 1 h. After washing three times with PBS-T, 100 μl of primary antibodies (1:10,000 in PBS) was added to each well, and incubated at 37°C for 1 h. After washing three times with PBS-T, 100 μl of peroxidase-conjugated goat anti-mouse IgG (H+L) (Yeasen, Shanghai, China) at a dilution of 1:5,000 in PBS was added to each well, and incubated at 37°C for 1 h. After washing five times with PBS-T, 100 μl 3,3′,5,5′-tetramethylbenzidine (Yeasen, Shanghai, China) was added to each well. Plates were kept in the dark for 5 min, and then 50 μl of 2.0 mol/l H_2_SO_4_ was added to stop the reaction. OD_450_ value was measured using a Microplate Reader (Sanjose, Shanghai, China). In addition, to determine if INP could bind to RE, INP BL21 was added to RE coated wells, followed by antibodies against INP (prepared as described in Li et al., 2009) and peroxidase-conjugated secondary antibody.

Romaine extract-coated or PGM-coated wells with PBS were used as the blank control. The bacteria *E. coli* BL21 was used as a negative control (N). Samples were considered as positive when the positive to genitive (P/N) ratio was greater than 2.0.

### Competitive Inhibition ELISA Assay

Boiled mixed saliva was diluted five times with PBS. The INP-P (GII.4) BL21 (100 μl) cells were incubated with the diluted boiled saliva (100 μl) or 1.0 mg/ml PGM (100 μl) solution at 37°C for 30 min. After centrifugation at 10,000 × *g* for 2 min at 4°C, the precipitated INP-P (GII.4) BL21 was re-suspended in PBS (pH = 7.4). The INP-P (GII.4) BL21 incubated with PBS was used as untreated control. *E. coli* BL21 (negative control) was treated with the same process. After blocking, the INP-P (GII.4) BL21 was detected by RE-binding based ELISA as described in the Section “Romaine Lettuce Extract-Binding or PGM-Binding Based ELISA”. To calculate percentage inhibition, the P/N ratio of untreated samples was equal to 0% inhibition and P/N ratio of 2.0 was equal to 100% inhibition.

### Protein denaturation or carbohydrate oxidation of LE and VE

Porcine gastric mucin was used as a positive control for protein denaturation and carbohydrate oxidation (Tian et al., 2005, 2007). LE or VE was boiled for 5 min, and centrifuged at 10,000 × *g* for 10 min at 4°C. The supernatant was diluted five times with carbonate buffer solution (pH = 9.6), and was used to coat the wells as described in Section “Romaine Lettuce Extract-Binding or PGM-Binding Based ELISA”. To determine whether carbohydrates were involved in INP-P (GII.4) BL21 binding, 100 μl 4.0 mg/ml NaIO_4_ (Adamas Reagent Co., Ltd., Shanghai, China) was added to wells coated with LE or VE (Esseili et al., 2012). The plates were incubated at 37°C for 30 min prior to the addition of the INP-P (GII.4) BL21. PGM, LE, or VE without protein denaturation or oxidation was used as untreated controls. After treatment, the binding capability and percentage of inhibition were evaluated as described in Sections “Romaine Lettuce Extract-Binding or PGM-Binding Based ELISA” and “Competitive Inhibition ELISA Assay”.

### The pH Effects on the Interaction between INP-P (GII.4) BL21 and Romaine Lettuce

The INP-P (GII.4) BL21 and *E. coli* BL21 (negative control) was diluted across a range of PBS of different pHs, and adjusted to the same OD_600_ value. The INP-P (GII.4) BL21 was detected by RE-binding-based ELISA as described in Section “Romaine Lettuce Extract-Binding or PGM-Binding Based ELISA”. P/N ratio at pH 6.4 was equal to 100% to calculate the percentage of binding.

### Statistics

Each experiment (N) was performed in triplicate (*n* = 3) and repeated at least three times (*N* > 3). The means and standard deviations from independent experiments were presented in all figures. One-way analysis of variance (ANOVA) was utilized for data comparison. Differences in means were considered significant when the *p* < 0.05.

## Results

### SD-GII.4P was Presented at the Surface of the Transformed *E. coli* BL21

**FIGURE 2:**
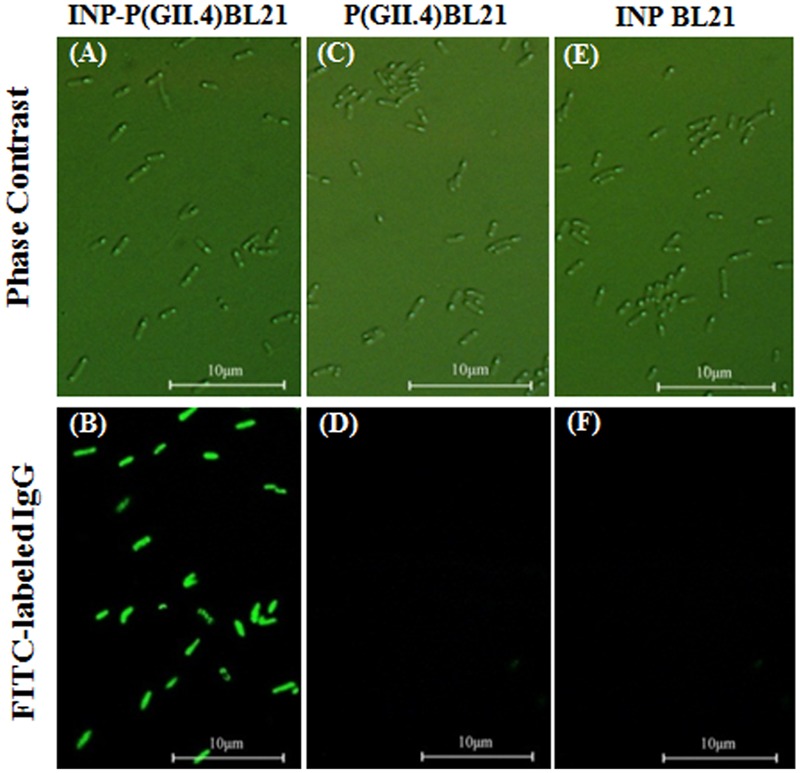
**Surface localization of SD-GII.4P.** INP-P (GII.4) BL21, P (GII.4) BL21, and INP BL21 were observed under phase contract microscopy **(A,C,E)**, and fluorescence microscopy **(B,D,F)**.

To verify the surface localization of SD-GII.4P on INP-P (GII.4) BL21 cells, immunofluorescence assay was performed (**Figure 2**). SD-GII.4P fusion proteins were clearly visualized as fluorescent spots on the transformed cell surface (**Figures 2A,B**). No FITC fluorescence signals were observed in P (GII.4) BL21 (**Figures 2C,D**), INP BL21 samples (**Figures 2E,F**) and *E. coli* BL21 (data not shown).

### Distribution of INP-P (GII.4) BL21 on Romaine Lettuce Surface

**FIGURE 3:**
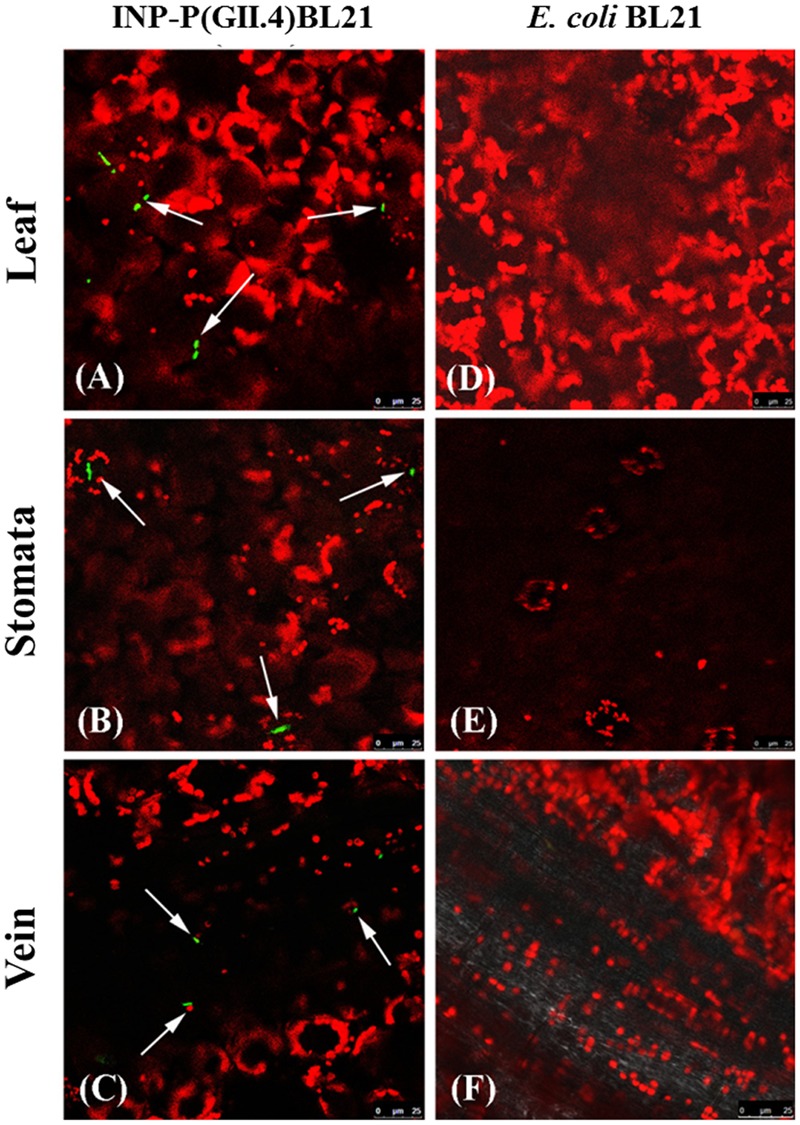
**Identification of SD-GII.4P binding to romaine lettuce by confocal immufluorescence assay.** Image (**A**, leaf), (**B**, stomata), and (**C**, vein) were from lettuce exposed to INP-P (GII.4) BL21s. Image (**D**, leaf), (**E**, stomata), and (**F**, vein) were from lettuce exposed to *E. coli* BL21. Green, INP-P (GII.4) BL21s; red, autofluorescence of chloroplasts. Arrows indicated the INP-P (GII.4) BL21s.

The INP-P (GII.4) BL21 bacteria were observed directly by Confocal Laser Scanning Microscope using FITC-labeled antibodies. After being washed, the INP-P (GII.4) BL21 bacteria were found to be distributed on the leaf (**Figure 3A**), stomata (**Figure 3B**) and vein (**Figure 3C**) of romaine lettuce. Romaine lettuce with *E. coli* BL21 bacteria exhibited no green fluorescent signals on its surface (**Figures 3D–F**). The red signals were autofluorescence of chloroplasts (**Figure 3**).

### INP-P (GII.4) BL21 Bound to LE and VE and the Binding Could Be Competitively Inhibited by HBGAs in Human Saliva

**FIGURE 4:**
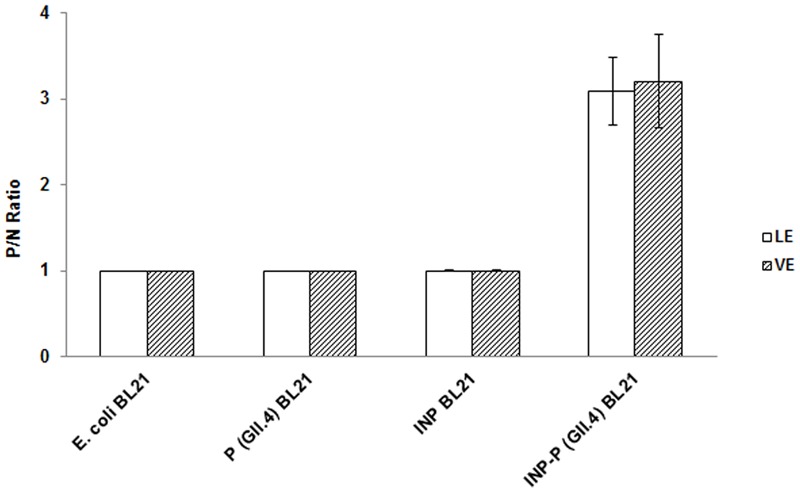
**Romaine extract (RE)-based ELISA assays of *E. coli* BL21, P (GII.4) BL21, INP BL21, and INP-P (GII.4) BL21.** LE, leaf extract; VE, vein extract. The means and standard deviations from independent experiments were presented. Error bars represent standard deviation.

**FIGURE 5:**
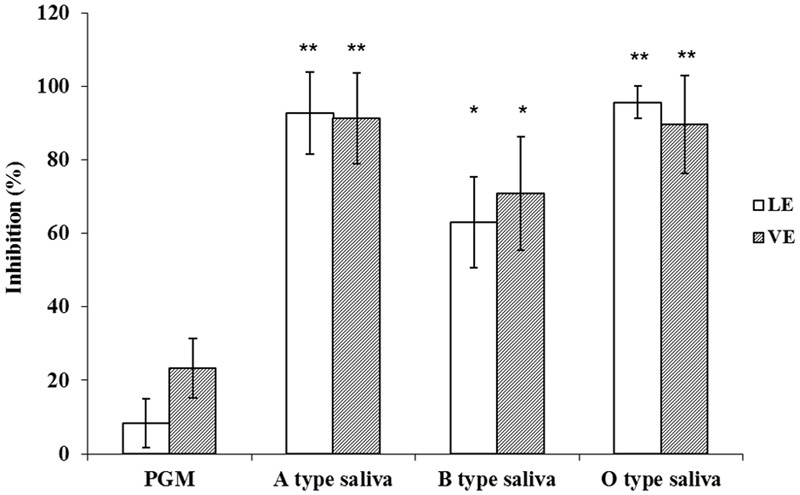
**Competitive inhibition ELISA assays.** Inhibition ability was shown as percentage. LE, leaf extract; VE, vein extract; PGM, porcine gastric mucin; A type saliva, B type saliva, and O type saliva indicated A, B, and O blood type saliva, respectively. The means and standard deviations from independent experiments were presented. Error bars represent standard deviation. Statistical differences of *p* < 0.05 were indicated by an asterisk and *p* < 0.01 were indicated by two asterisks.

The OD_450_ reading of *E. coli* BL21 was used as control N to calculate P/N ratio in RE binding based ELISA and inhibition assays. The P/N ratio of P (GII.4) BL21 and INP BL21 were all around 1.0 for LE and VE with antibodies against VP1 of GII.4 HuNoV (**Figure 4**). There were no significant differences between these two samples and *E. coli* BL21. The result suggested that BL21 and InaQN could not bind to RE measured by HuNoV specific antibodies. In addition, with InaQN specific antibodies, we demonstrated that INP BL21 could not bind to RE coated wells as its P/N ratio was closed to *E. coli* BL21 control (data not shown). We further demonstrated that HuNoV P proteins expressed in P (GII.4) BL21 were not be able to bind RE due to its nature of intracellular expression. However, INP-P (GII.4) BL21 could bind to wells coated with LE or VE. The P/N ratios ranged from 2.38 to 4.01 for LE and 2.40 to 4.21 for VE. A slight stronger but not significant binding of INP-P (GII.4) BL21 was observed in VE (3.21 ± 0.92) than that of LE (3.09 ± 0.84). The average inhibition rates were 92.68 ± 11.27%, 95.64 ± 4.46%, 62.98 ± 12.25%, and 8.31 ± 6.57% when INP-P (GII.4) BL21 was pre-incubated with type A, O, B saliva, or PGM (**Figure 5**) prior to being added to LE-coated wells. Similarly, the average inhibition rates were 91.32 ± 12.31%, 89.55 ± 13.37%, 70.80 ± 15.37%, and 23.36 ± 8.11% when INP-P (GII.4) BL21 was pre-incubated with type A, O, B saliva, or PGM prior to being added to VE-coated wells (**Figure 5**). The binding of INP-P (GII.4) BL21 to both LE and VE could be completely inhibited by type-A and -O saliva, and partially inhibited by PGM and type-B saliva. There was no significant difference between the binding of INP-P (GII.4) BL21 to leaf and vein areas of romaine lettuce. In addition, the binding between INP-P (GII.4) BL21 and LE or VE could not be inhibited by saliva from non-secretors (data not shown).

### Characterization of Components in Romaine Lettuce Binding with INP-P (GII.4) BL21

**FIGURE 6:**
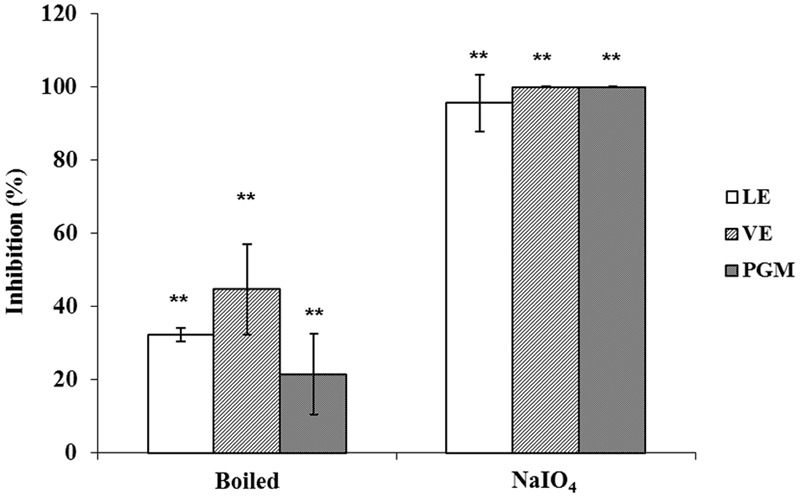
**Protein denaturation and carbohydrate oxidation of LE, VE, and PGM.** Inhibition ability was shown as percentage. LE, leaf extract; VE, vein extract; PGM, porcine gastric mucin. The means and standard deviations from independent experiments were presented. Error bars represented standard deviation. Statistical differences of *p* < 0.01 were indicated by two asterisks.

To determine the nature of the receptor/ligand of romaine lettuce involved in binding INP-P (GII.4) BL21, LE, and VE were either boiled to denature proteins, or oxidized by NaIO_4_ to remove the functional groups of carbohydrates. It has been reported that HBGAs in PGM were involved in the binding of HuNoV (Tian et al., 2005). Therefore, PGM was used as positive control for the assay. The effects of protein denaturation and carbohydrate oxidation on LE and VE were similar to that of the same to PGM. Wells coated with NaIO_4_-oxidized LE, VE or PGM resulted in complete inhibition of binding with INP-P (GII.4) BL21 with inhibition rates of 95.49 ± 7.81%, 100%, and 100%, respectively (**Figure 6**). A significant difference in binding inhibition was observed between NaIO_4_-treated and untreated reactions with the three groups, but no significant difference was observed between the three groups (*p* > 0.05). Partial inhibition was observed when INP-P (GII.4) BL21 was applied to wells coated with LE, VE or PGM treated with heat to denature proteins (**Figure 6**). When PGM, LE and VE were heat-treated the inhibition rates were 21.48 ± 11.09%, 32.23 ± 1.76%, and 44.66 ± 12.36%, respectively. There was a significant difference between these three groups treated with or without heat-treatment but no significant difference among these three groups (*p* > 0.05). These results demonstrated that carbohydrates rather than proteins played an important role in LE and VE for HuNoV binding.

### Effect of pH on the Binding of INP-P (GII.4) BL21 to LE, VE, and PGM

**FIGURE 7:**
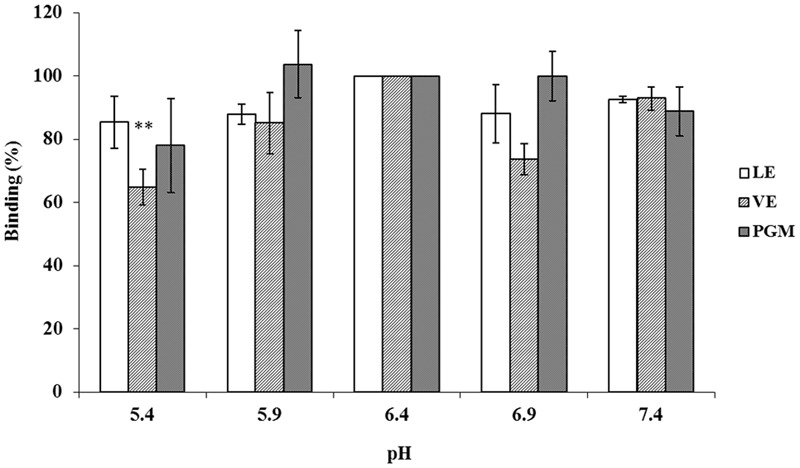
**The pH effects on the interaction between INP-P (GII.4) BL21s and romaine lettuce.** Binding ability was shown as percentage. LE, leaf extract; VE, vein extract; PGM, porcine gastric mucin. The means and standard deviations from independent experiments were presented. Error bars represented standard deviation. Statistical differences of *p* < 0.01 were indicated by two asterisks.

To determine the effect of pH on the binding of INP-P (GII.4) BL21 bacteria to romaine lettuce, especially in the vicinity of isoelectric point (pI) of the P domain protein (pI = 6.4), LE or VE were treated at conditions below, above, and at pI of P protein (**Figure 7**). PGM was used as a control. Aside from a significant reduction of binding between INP-P (GII.4) BL21 to VE at pH 5.4 (*p* < 0.004), there was no significant difference between binding of INP-P (GII.4) BL21 bacteria to PGM, LE, or VE at various pHs tested.

## Discussion

Contaminated fresh produce is one of the main causes of HuNoV infections around the world (Ethelberg et al., 2010). However, the mechanisms of interactions between viruses and fresh produce are poorly understood. As the virus cannot be easily cultured *in vitro*, it is difficult for many labs to collect enough HuNoVs to perform direct studies. Although VLPs and P particles were developed as surrogates for HuNoV, the preparation of these surrogates are themselves complicated. Making VLPs from recombinant eukaryotic systems is both complicated and time-consuming. P particles could be produced in a prokaryotic system, but are expressed intracellularly and therefore require purification, enzyme digestion, and re-purification steps. In addition, the small sizes of VLPs and P particles make microscopic observation difficult, and low-speed centrifugation isolation of the viral protein-ligand/receptor complex impossible. In this study, we demonstrated that the P proteins expressed by P (GII.4) BL21 could not be expressed on the surface of bacteria and could not bind to RE extracts (**Figures 2** and **4**). Therefore, we engineered a novel system to present HuNoV VP1 or P proteins on the surface of transformed bacteria (Niu et al., 2015). The new system uses ice nucleation protein (INP)-target fusion protein to display the target protein on the surface of transformed *E. coli* BL21. It has been shown that by transforming bacteria with a gene encoding for a fusion target protein with the anchoring motifs of INP, the target protein could be directly displayed on the surface of the bacteria (Cochet and Widehem, 2000; Li et al., 2009, 2012). The advantages of this surface display system are: (1) compared with viruses, bacteria are easy to culture, collect and observe under microscope. (2) Compared with VLPs or P particles, this surface display system can allow protein products to interact with receptors/ligands directly, requiring neither cell disruption nor protein purification steps. In our previous study, we demonstrated that SD-GII.4VP1 and SD-GII.4P could recognize and bind HBGAs (Niu et al., 2015). SD-GII.4P showed a better HBGA-binding ability than that of SD-GII.4VP1 and was selected for use in this study as a model to determine the interactions between the HuNoVs and romaine lettuce.

Surface localization of SD-GII.4P was confirmed by immunofluorescence assay (**Figure 2**). The SD-GII.4P could be found on the leaf surfaces, stomata and veins of romaine lettuce (**Figure 3**). The results were consistent with previous reports. Gandhi et al. (2010) reported that GI.1 VLPs could be found in clusters along the veins. Esseili et al. (2012) reported that GII.4 VLPs bound to leaves of romaine lettuce. Finding SD-GII.4P in stomata in this study is interesting as stomata might provide protection to viruses from liquid disinfectants or simple washing. It is common to find bacteria in stomata because of the move ability of bacteria (Xicohtencatl-Cortes et al., 2009; Golberg et al., 2011; Kroupitski et al., 2011). Wei et al reported that MNV could be found in stomata, at the cut edges and on leaf surfaces of romaine lettuce (Wei et al., 2010). DiCaprio et al reported that HuNoV VLPs, TV, and MNV could be found in stomata (Dicaprio et al., 2012). It remains unknown when and how these viruses/VLPs got into stomata, and their entry with or without binding to bacteria. In this study, we reported that SD-GII.4P could get into stomata *via* bacteria. As some enteric bacteria are capable of expressing HBGAs that bind HuNoVs, it raises the possibility that enteric bacteria might facilitate the entry of HuNoVs into stomata, which confers protection from washing and disinfection processes. This could occur by either HuNoVs binding to bacterial HBGAs prior to the latter’s entry into stomata, or binding to bacteria already in stomata.

Vega et al used four different viruses to determine the effect of pH on viral attachment to lettuce (Vega et al., 2005). The results showed that the pI of viruses was not the governing factor in their attachment to lettuce. Similarly, our results also indicated that the pI of P protein was not a major factor in the binding between INP-P (GII.4) BL21 and lettuce (**Figure 7**). As the bacteria were not stable in a lower or higher pH, only pH around the pI of P domain were tested. Although no charge exists on the surface of P protein at pI (pH 6.4), INP-P (GII.4) BL21 still exhibits a binding ability to RE, suggesting a specific binding between receptor and ligand. To determine if specific molecules in romaine lettuce were involved in the binding of SD-GII.4P, a competitive binding assay was performed (**Figure 5**). The binding of SD-GII.4P to LE and VE could be completely inhibited by human saliva containing type-A or -O HBGAs, and could be partially inhibited by human saliva containing type-B HBGAs. The binding ability of LE and VE could also be abolished when their carbohydrates are oxidized by NaIO_4_ (**Figure 6**). All these results suggest that type-A and -O HBGAs-like molecules in LE and VE were responsible for HuNoV P protein binding. Our results were consistent with reports from other groups. Gao et al reported that monoclonal antibodies against HBGAs could inhibit the interaction between the GII.4 VLPs and lettuce leaf (Gao et al., 2016). Esseili et al. (2012) reported that multiple carbohydrate moieties, mainly α-D-Gal, GalNAc, Man/Glc, Fuc, GlcNAc, and sialic acid, were involved in the binding of GII.4 NoV VLPs to leaf cell wall materials of romaine lettuce. Gandhi et al. (2010) reported that GI.I VLPs could bind to romaine lettuce veins and this binding could not be completely inhibited by PGM. Similarly, our results also indicated that PGM could not completely inhibit the binding of SD-GII.4P to LE and VE. It is possible that HBGAs present in human saliva was much higher than that of PGM used in the study.

It was reported that the isolated P protein could spontaneously dimerize, and stabilized the protein in solution. In addition, the P dimers could also combine to form P particles, which were composed of 12 sets of P dimers. Both the P dimer and P particle were capable of binding HBGAs (Tan and Jiang, 2005; Cao et al., 2007). Gao et al reported that both A and B trisaccharides were located near the P dimer interface, suggesting that dimerization was both important for structural stability, and essential for its receptor binding functionality (Gao et al., 2016). It remains unknown if HBGAs could bind to P protein monomers. In our study, the bacterial-surface-displayed P protein had the ability to interact with HBGA-like molecules from romaine lettuce. It remains unknown if SD-GII.4P remains monomers or forms dimers on the surface of transformed bacteria to bind HBGA-like molecules in romaine lettuce. Currently, we are in the process of investigating whether the P monomers or dimers on the bacterial surface participate in the binding of HBGAs.

In this study, we showed that INP-P (GII.4) BL21 could bind to HBGA-like molecules in leaves and veins of romaine lettuce. The nature of the HBGA-like ligand in romaine lettuce remains unknown. Currently, we are in the process of developing protocols to separate the SD-GII.4P-ligand complex in order to release the ligands for characterization.

## Conclusion

The interaction between GII.4 HuNoV and ligand candidates in romaine lettuce was confirmed by using a novel bacterial expression system that expressed P protein of GII.4 HuNoV on the surface of transformed bacteria. This new yet simple surface display system could be used in the future for the purpose of isolating and characterizing receptor/ligand candidates for HuNoVs.

## Author Contributions

LQ, WD, and RS designed the experiments. WM carried out the experiments with assistance from RS, ZY, GS, and WD. RS, WM, TP, and LQ conducted statistical analysis. WM, TP, and LQ wrote the paper. WD modified the paper. All authors reviewed the results, made substantial contributions and approved the final version of the manuscript.

## Conflict of Interest Statement

The authors declare that the research was conducted in the absence of any commercial or financial relationships that could be construed as a potential conflict of interest.

## References

[B1] Bozkurt H. D’Souza D. H. Davidson P. M. (2013). Determination of the thermal inactivation kinetics of the human norovirus surrogates, murine norovirus and feline calicivirus. *J. Food Prot.* 76 79–84. 10.4315/0362-028X.JFP-12-327 23317860

[B2] Cao S. Lou Z. Tan M. Chen Y. Liu Y. Zhang Z. (2007). Structural basis for the recognition of blood group trisaccharides by norovirus. *J. Virol.* 81 5949–5957. 10.1128/JVI.00219-07 17392366 PMC1900264

[B3] Cochet N. Widehem P. (2000). Ice crystallization by *Pseudomonas syringae*. *Appl. Microbiol. Biotechnol.* 54 153–161. 10.1007/s002530000377 10968626

[B4] Dicaprio E. Ma Y. Purgianto A. Hughes J. Li J. (2012). Internalization and dissemination of human norovirus and animal caliciviruses in hydroponically grown romaine lettuce. *Appl. Environ. Microbiol.* 78 6143–6152. 10.1128/AEM.01081-12 22729543 PMC3416640

[B5] Dicaprio E. Purgianto A. Ma Y. Hughes J. Dai X. Li J. (2015). Attachment and localization of human norovirus and animal caliciviruses in fresh produce. *Int. J. Food Microbiol.* 211 101–108. 10.1016/j.ijfoodmicro.2015.07.013 26188496

[B6] Esseili M. A. Wang Q. Saif L. J. (2012). Binding of human GII.4 norovirus virus-like particles to carbohydrates of romaine lettuce leaf cell wall materials. *Appl. Environ. Microbiol.* 78 786–794. 10.1128/AEM.07081-11 22138991 PMC3264112

[B7] Ethelberg S. Lisby M. Bottiger B. Schultz A. C. Villif A. Jensen T. (2010). Outbreaks of gastroenteritis linked to lettuce, Denmark, January 2010. *Euro Surveill.* 15:19484. 20158982

[B8] Ettayebi K. Crawford S. E. Murakami K. Broughman J. R. Karandikar U. Tenge V. R. (2016). Replication of human noroviruses in stem cell-derived human enteroids. *Science* 353 1387–1393. 10.1126/science.aaf5211 27562956 PMC5305121

[B9] Gandhi K. M. Mandrell R. E. Tian P. (2010). Binding of virus-like particles of Norwalk virus to romaine lettuce veins. *Appl. Environ. Microbiol.* 76 7997–8003. 10.1128/AEM.01566-10 21037300 PMC3008227

[B10] Gao X. Esseili M. A. Lu Z. Saif L. J. Wang Q. (2016). Recognizing HBGA-like carbohydrates in lettuce by human GII.4 norovirus. *Appl. Environ. Microbiol.* 82 2966–2974. 10.1128/AEM.04096-15 26969699 PMC4959087

[B11] Golberg D. Kroupitski Y. Belausov E. Pinto R. Sela S. (2011). *Salmonella* Typhimurium internalization is variable in leafy vegetables and fresh herbs. *Int. J. Food Microbiol.* 145 250–257. 10.1016/j.ijfoodmicro.2010.12.031 21262550

[B12] Grove S. F. Suriyanarayanan A. Puli B. Zhao H. Li M. Li D. (2015). Norovirus cross-contamination during preparation of fresh produce. *Int. J. Food Microbiol.* 198 43–49. 10.1016/j.ijfoodmicro.2014.12.023 25590260

[B13] Hoa Tran T. N. Trainor E. Nakagomi T. Cunliffe N. A. Nakagomi O. (2013). Molecular epidemiology of noroviruses associated with acute sporadic gastroenteritis in children: global distribution of genogroups, genotypes and GII.4 variants. *J. Clin. Virol.* 56 185–193. 10.1016/j.jcv.2012.11.011 23218993

[B14] Jiang X. Wang M. Graham D. Y. Estes M. K. (1992). Expression, self-assembly, and antigenicity of the Norwalk virus capsid protein. *J. Virol.* 66 6527–6532. 1328679 10.1128/jvi.66.11.6527-6532.1992 PMC240146

[B15] Jones M. K. Grau K. R. Costantini V. Kolawole A. O. de Graaf M. Freiden P. (2015). Human norovirus culture in B cells. *Nat. Protoc.* 10 1939–1947. 10.1038/nprot.2015.121 26513671 PMC4689599

[B16] Kroupitski Y. Pinto R. Belausov E. Sela S. (2011). Distribution of *Salmonella* typhimurium in romaine lettuce leaves. *Food Microbiol.* 28 990–997. 10.1016/j.fm.2011.01.007 21569943

[B17] Li Q. Yan Q. Chen J. He Y. Wang J. Zhang H. (2012). Molecular characterization of an ice nucleation protein variant (inaQ) from *Pseudomonas syringae* and the analysis of its transmembrane transport activity in *Escherichia coli*. *Int. J. Biol. Sci.* 8 1097–1108. 10.7150/ijbs.4524 22991498 PMC3445048

[B18] Li Q. Yu Z. Shao X. He J. Li L. (2009). Improved phosphate biosorption by bacterial surface display of phosphate-binding protein utilizing ice nucleation protein. *FEMS Microbiol. Lett.* 299 44–52. 10.1111/j.1574-6968.2009.01724.x 19686343

[B19] Made D. Trubner K. Neubert E. Hohne M. Johne R. (2013). Detection and typing of norovirus from frozen strawberries involved in a large-scale gastroenteritis outbreak in Germany. *Food Environ. Virol.* 5 162–168. 10.1007/s12560-013-9118-0 PMC3753465 23888384

[B20] Mok H. F. Barker S. F. Hamilton A. J. (2014). A probabilistic quantitative microbial risk assessment model of norovirus disease burden from wastewater irrigation of vegetables in Shepparton, Australia. *Water Res.* 54 347–362. 10.1016/j.watres.2014.01.060 24594660

[B21] Niu M. Yu Q. Tian P. Gao Z. Wang D. Shi X. (2015). Engineering bacterial surface displayed Human norovirus capsid proteins: a novel system to explore interaction between norovirus and ligands. *Front. Microbiol.* 6:1448 10.3389/fmicb.2015.01448 PMC4686607 26733983

[B22] Sarvikivi E. Roivainen M. Maunula L. Niskanen T. Korhonen T. Lappalainen M. (2012). Multiple norovirus outbreaks linked to imported frozen raspberries. *Epidemiol. Infect.* 140 260–267. 10.1017/S0950268811000379 21418716

[B23] Tamminen K. Huhti L. Koho T. Lappalainen S. Hytönen V. P. Vesikari T. (2012). A comparison of immunogenicity of norovirus GII-4 virus-like particles and P-particles. *Immunology* 135 89–99. 10.1111/j.1365-2567.2011.03516.x 22044070 PMC3246655

[B24] Tan M. Fang P. Chachiyo T. Xia M. Huang P. Fang Z. (2008). Noroviral P particle: structure, function and applications in virus-host interaction. *Virology* 382 115–123. 10.1016/j.virol.2008.08.047 18926552 PMC3508508

[B25] Tan M. Fang P. Xia M. Chachiyo T. Jiang W. Jiang X. (2011). Terminal modifications of norovirus P domain resulted in a new type of subviral particles, the small P particles. *Virology* 410 345–352. 10.1016/j.virol.2010.11.017 21185050 PMC3064930

[B26] Tan M. Jiang X. (2005). The p domain of norovirus capsid protein forms a subviral particle that binds to histo-blood group antigen receptors. *J. Virol.* 79 14017–14030. 10.1128/JVI.79.22.14017-14030.2005 16254337 PMC1280206

[B27] Tian P. Brandl M. Mandrell R. (2005). Porcine gastric mucin binds to recombinant norovirus particles and competitively inhibits their binding to histo-blood group antigens and Caco-2 cells. *Lett. Appl. Microbiol.* 41 315–320. 10.1111/j.1472-765X.2005.01775.x 16162137

[B28] Tian P. Jiang X. Zhong W. Jensen H. M. Brandl M. Bates A. H. (2007). Binding of recombinant norovirus like particle to histo-blood group antigen on cells in the lumen of pig duodenum. *Res. Vet. Sci.* 83 410–418. 10.1016/j.rvsc.2007.01.017 17379264

[B29] Vega E. Smith J. Garland J. Matos A. Pillaii S. D. (2005). Variability of virus attachment patterns to butterhead lettuce. *J. Food Prot.* 68 2112–2117. 16245715 10.4315/0362-028x-68.10.2112

[B30] Verhaelen K. Bouwknegt M. Lodder-Verschoor F. Rutjes S. A. de Roda Husman A. M. (2012). Persistence of human norovirus GII.4 and GI.4, murine norovirus, and human adenovirus on soft berries as compared with PBS at commonly applied storage conditions. *Int. J. Food Microbiol.* 160 137–144. 10.1016/j.ijfoodmicro.2012.10.008 23177054

[B31] Vinjé J. (2015). Advances in laboratory methods for detection and typing of norovirus. *J. Clin. Microbiol.* 53 373–381. 10.1128/JCM.01535-14 24989606 PMC4298492

[B32] Wang D. Xu S. Yang D. Young G. M. Tian P. (2014). New *in situ* capture quantitative (real-time) reverse transcription-PCR method as an alternative approach for determining inactivation of Tulane virus. *Appl. Environ. Microbiol.* 80 2120–2124. 10.1128/AEM.04036-13 24463967 PMC3993159

[B33] Wang Q. Zhang Z. Saif L. J. (2012). Stability of and attachment to lettuce by a culturable porcine sapovirus surrogate for human caliciviruses. *Appl. Environ. Microbiol.* 78 3932–3940. 10.1128/AEM.06600-11 22447610 PMC3346393

[B34] Wei J. Jin Y. Sims T. Kniel K. E. (2010). Manure- and biosolids-resident murine norovirus 1 attachment to and internalization by Romaine lettuce. *Appl. Environ. Microbiol.* 76 578–583. 10.1128/AEM.02088-09 19933344 PMC2805210

[B35] Xicohtencatl-Cortes J. Sanchez Chacon E. Saldana Z. Freer E. Giron J. A. (2009). Interaction of *Escherichia coli* O157:H7 with leafy green produce. *J. Food Prot.* 72 1531–1537. 19681282 10.4315/0362-028x-72.7.1531

[B36] Xu S. Wang D. Yang D. Liu H. Tian P. (2015). Alternative methods to determine infectivity of Tulane virus: a surrogate for human nororvirus. *Food Microbiol.* 48 22–27. 10.1016/j.fm.2014.12.004 25790987

